# Perceived personal importance of exercise and fears of re-injury: a longitudinal study of psychological factors related to activity after anterior cruciate ligament reconstruction

**DOI:** 10.1186/2052-1847-7-4

**Published:** 2015-01-21

**Authors:** Monique AM Gignac, Xingshan Cao, Subha Ramanathan, Lawrence M White, Mark Hurtig, Monica Kunz, Paul H Marks

**Affiliations:** Institute for Work and Health; Division of Health Care & Outcomes Research, Arthritis Community Research and Evaluation Unit, Toronto Western Research Institute; Dalla Lana School of Public Health, University of Toronto, Toronto, Canada; Arthritis Community Research and Evaluation Unit, Toronto Western Research Institute, Toronto, Canada; Postdoctoral Fellow, Faculty of Kinesiology and Physical Education, University of Toronto, Toronto, Canada; Joint Department of Medical Imaging, Faculty of Medicine, University Health Network, Mount Sinai Hospital, Women’s College Hospital, University of Toronto, Toronto, Canada; Ontario Veterinary College, University of Guelph, Guelph, Canada; Division of Orthopaedic Surgery, Sunnybrook Health Sciences Centre, Toronto, Canada; Division of Orthopaedic Surgery, Faculty of Medicine, Sunnybrook Health Sciences Centre, University of Toronto, Toronto, Canada

**Keywords:** Physical activity, Sports injuries, Anterior cruciate ligament reconstruction, ACL injuries, Fear of re-injury, Perceived importance of exercise, Exercise identity, Psychological factors, Osteoarthritis

## Abstract

**Background:**

Psychological perceptions are increasingly being recognized as important to recovery and rehabilitation post-surgery. This research longitudinally examined perceptions of the personal importance of exercise and fears of re-injury over a three-year period post anterior cruciate ligament (ACL) reconstruction. Stability and change in psychological perceptions was examined, as well as the association of perceptions with time spent in different types of physical activity, including walking, household activities, and lower and higher risk for knee injury activities.

**Methods:**

Participants were athletes, 18–40 years old, who underwent ACL reconstruction for first-time ACL injuries. They were recruited from a tertiary care centre in Toronto, Canada. Participants completed interviewer-administered questionnaires pre-surgery and at years one, two and three, postoperatively. Questions assessed demographics, pain, functional limitations, perceived personal importance of exercise, fear of re-injury and physical activities (i.e., walking; household activities; lower risk for knee injury activities; higher risk for knee injury activities). Analyses included fixed-effect longitudinal modeling to examine the association of a fear of re-injury and perceived personal importance of exercise and changes in these perceptions with the total hours spent in the different categories of physical activities, controlling for other factors.

**Results:**

Baseline participants were 77 men and 44 women (mean age = 27.6 years; SD = 6.2). At year three, 78.5% of participants remained in the study with complete data. Fears of re-injury decreased over time while personal importance of exercise remained relatively stable. Time spent in walking and household activities did not significantly change with ACL injury or surgery. Time spent in lower and higher risk of knee injury physical activity did not return to pre-injury levels at three years, post-surgery. Greater time spent in higher risk of knee injury activities was predicted by decreases in fears of re-injury and by greater personal importance of exercise.

**Conclusions:**

This study highlights not only fears of re-injury, which has been documented in previous studies, but also the perceived personal importance of exercise in predicting activity levels following ACL reconstructive surgery. The findings can help in developing interventions to aid individuals make decisions about physical activities post knee injury and surgery.

## Background

There is considerable interest in the role that psychological perceptions play in understanding whether individuals resume physical activities after a sports injury. This is largely due to evidence finding that physical recovery from an injury and psychological readiness to return to activities like sports do not always correspond [1–4]. Specifically, many individuals do not return to pre-injury levels of activity despite good surgical or rehabilitation outcomes [1, 4–7]. Anterior cruciate ligament (ACL) injuries are a common cause for surgical intervention in sports medicine [8–10]. Estimates vary but suggest that, although 80% of individuals return to some type of sports by two years post ACL surgery, only about one to two thirds return to previous levels of activity [1, 4].

Common factors identified with lower levels of sport or physical activity post-ACL reconstruction are fears of re-injury and ongoing knee functional limitations [1–3, 11–17]. However, studies examining fear of re-injury often have been cross-sectional, have focused on competitive sports in small samples of athletes or have been limited to the first 12 months post ACL reconstruction [1–4, 12]. Fewer studies examine psychological perceptions over time, particularly changes in perceptions and their association with the type or level of activities.

In addition, there is little research on the personal importance of exercise to those who have sustained an ACL injury [11] despite studies finding that individuals for whom exercise is an important part of their identity are more likely to adopt and maintain physical activity [18–21]. This suggests that individuals with high personal importance of exercise may be more likely to resume previous levels of activity after ACL reconstruction. However, it is unclear what relationship exists between personal importance of exercise and fears of re-injury and whether, over time, higher exercise importance counteracts fears of re-injury and is associated with greater activity levels.

This study examined perceptions of the importance of exercise and fears of re-injury pre-surgery and at three year intervals post-ACL surgery. We examined whether perceptions were relatively stable or whether they changed post-surgery, as well as whether they were associated with physical activity over time, controlling for age, sex, body mass index (BMI), pain, and functional limitations [1–7, 12, 22–27]. Rather than focus on a single sport, we examined a range of activities [1, 7]. Of interest was whether individuals would be less likely to return to potentially higher risk for knee injury physical activity than lower risk for knee injury physical activity, walking, and household activities. Research suggests that activities and sports that involve contact, pivoting, and greater loads on the knee (e.g., soccer, basketball, football) are more prone to ACL injuries and represent higher risk of knee injury activities [10, 11, 28, 29]. Individuals may have greater fears of re-injury related to these types of activities and be less likely to engage in them. However, higher perceived importance of exercise may counteract these fears and be associated with greater time spent in higher risk of knee injury activities.

## Methods

### Participants

Respondents were recruited from April 2005- June 2007 in a large, tertiary care centre in Toronto, Canada. Participants experienced a first-time ACL injury and were between 18–40 years old. Male and female recreational, amateur and professional athletes were eligible for participation. Individuals were excluded if they had injured their ACL more than 6 weeks prior to their hospital visit or had reconstructive surgery scheduled for >3 months after their surgical intake interview. Patients also were excluded if they had a previous ACL injury or reconstruction, exhibited other bone pathology, pre-existing arthritis, or other systemic diseases. ACL reconstruction procedures were performed by one surgeon (PM) using a single incision, arthroscopic bone patellar tendon bone, transtibial reconstruction. All concomitant intra-articular pathologies were dealt with at the time of surgery (e.g., meniscal tears, plica, cartilage lesions). Femoral sided ACL graft fixation was performed with two bioabsorbable crosspins through the bone plug fixed with the knee at 90 degrees. A metal press fit interference screw was placed to secure the tibial side with the knee at 20–30 degrees flexion and with a posterior directed force on the anterior tibia and counterforce through stay sutures in the tibial bone plug. On occasion, there was graft size mismatch resulting in a longer graft which required tibial fixation using a trough with press fit fixation of the bone plug and a staple over top. Patients underwent a nine-month rehabilitation program with standard protocols. Regular rehabilitation appointments were held (e.g., postoperatively; 2–4 weeks; 5–10 weeks; 3–4 months; 4–8 months; > 9 months). Rehabilitation included positioning and ambulation assessments (e.g., use of splints/braces), exercise activities that built toward sport-specific drills; and shared patient-practitioner goals for reducing swelling and pain, increasing range of motion, flexibility and cardiovascular fitness. Information about the study, screening for eligibility and informed consent occurred when patients attended a pre-surgical visit. Ethics approval was received from Sunnybrook Hospital, Toronto, Canada and informed, written consent was obtained from all participants.

### Procedure

This study uses baseline and years one, two and three follow-up questionnaire data from a five-year longitudinal study examining outcomes from ACL reconstructive surgery. Participants completed a standard set of interviewer-administered questionnaires and provided clinical data for the study. Questionnaires were administered by a trained research assistant in a private room at the hospital and took approximately 45 minutes to complete. Baseline questionnaires were administered during a preoperative visit that was, on average, 40 days post-injury. Questionnaires were re-administered annually (Year one, Year two, Year three) postoperatively.

### Measures

#### Physical Activity

The *Minnesota Leisure-time Physical Activity Questionnaire* (MLPAQ) assessed the type, frequency and intensity of involvement in different activities over a 12-month period [30]. The questionnaire asks about 54 different physical activities related to walking, conditioning, individual and team sports, racquet sports, water activities, winter activities, housework, home repair, and caregiving. It has been widely used and validated in epidemiological studies and interventions [31–34]. Participants can add activities if not listed in the questionnaire. For each activity, participants were asked whether they performed the activity and, if so, the average times per month and average minutes spent on each occasion. Using this, the total number of hours in different activities over the last year was calculated. Physical activities were grouped into three broad categories: household activities, walking activities, and sports and leisure physical activities. Caregiving activities (i.e., lifting a dependent) were omitted. Drawing on previous studies, sports and leisure activities were further divided into two groups: lower risk of knee injury and higher risk of knee injury activities [10, 11, 28, 29]. Higher risk of knee injury activities were defined as contact sports, pivoting sports, activities with greater ground forces on landing or loads on the knee, and the potential for greater knee abduction or hyperextension. If an individual did not engage in any activities within a category they were assigned a score of zero hours in that activity. Two coders classified the activities independently. Inter-rater reliability for assignment into the two categories was high (kappa = .88) (Table 1).

**Table 1:** **Examples of lower and higher risk of knee injury physical activities reported by respondents**

Lower risk of knee injury physical activities	Higher risk of knee injury physical activities
Slow-pitch	Football
Pilates	Beach volleyball
Yoga	Rugby
Body sculpting	Gymnastics
Skipping	Lacrosse
Tai Chi	Cliff jumping
Kayaking	Hockey
Canoeing	Wrestling
White water rafting	Mountain biking
Physiotherapy exercises	Squash/racquetball
Golf	Trampoline
Elliptical training	Field hockey
Snow shoeing	Snowboarding
Stair climber	Martial arts
Paint ball	Tennis
Cycling	Waterskiing
Swimming	Soccer
Jogging	Ultimate Frisbee
Sailing	Basketball
Scuba diving	
Cross country skiing	
Tobogganing	

#### Fear of re-injury

The previously validated *Anterior Cruciate Ligament – Quality of Life (ACL-QOL)* questionnaire measured perceptions of a fear of re-injury in the previous three months with a single item [35]. Participants were asked, “How fearful are you of reinjuring your knee?” Responses ranged from 0 = extremely fearful to 100 = no fear at all. Scores were reverse coded with higher values indicating greater fear of re-injury.

#### Perceived Personal Importance of Exercise

Perceptions of the personal importance of exercise to respondents were measured with a validated 9-item *Exercise Identity Scale*[36]. Sample items included, “I consider myself an exerciser” and “I need to exercise to feel good about myself.” Responses were on a 5-point Likert-type scale from 1 = strongly disagree to 5 = strongly agree. Total scores were calculated by summing items. Higher scores reflected greater importance of exercise. Cronbach’s alphas, a measure of the internal consistency of items, ranged from 0.91-0.93 across the different time-points of data collection.

#### Pain

Nine items from the pain subscale of the *Knee Injury and Osteoarthritis Outcome Score* (KOOS) assessed the frequency and degree of pain in the previous week [37]. The KOOS has been widely used and validated [38]. Items examined pain associated with twisting/pivoting, walking on a flat surface, and going up/down stairs. Scores ranged from 0–100 with lower scores reflecting greater pain with activities [39]. Cronbach’s alphas for the measure ranged from 0.82-0.87 across the time-points.

#### Sports and recreation activity limitations

Functional limitations in sports and recreational activities were measured with 12 items from the recreational activities and sport participation subscale of the *Anterior Cruciate Ligament – Quality of Life (ACL-QOL)* questionnaire [35]. Responses were assessed over the previous three months on a visual analogue scale with an anchor of zero reflecting greater limitations, difficulties and concerns and an anchor of 100 indicating no limitations, difficulties or concerns. A total sports and recreational functional limitations score was calculated [40–43]. Cronbach’s alphas ranged from 0.78-0.95.

#### Demographic information

Age, gender and type of sport in which the injury occurred were collected at baseline. Data for height and weight were collected annually and used to calculate Body Mass Index (BMI) scores.

### Analyses

Sample descriptive statistics were calculated at baseline and at follow-ups, including frequencies, means, and standard deviations. Cross-wave comparisons examined means and 95% confidence intervals (CI’s) for the time spent in different categories of physical activity (i.e., walking, household activities, lower risk of knee injury activity, higher risk of knee injury activity) at baseline and Year one to three follow-up time-points. Comparisons across time also were made using means and 95% CI’s for fear of re-injury; personal importance of exercise; pain; sports and recreational activity limitations; and BMI. Pearson correlation coefficients examined the relationship between fear of re-injury and perceived exercise identity. To further describe individual differences in hours spent in lower and higher risk for knee injury physical activities at the Year three follow-up, respondents were divided into four groups based on median values for fear of re-injury and perceived personal importance of exercise (low fear of re-injury/low personal importance of exercise; low fear of re-injury/high personal importance of exercise; high fear of re-injury/low personal importance of exercise; high fear of re-injury/high personal importance of exercise). Year three hours of lower and higher risk for knee injury physical activity were compared to baseline time spent in these activities across the groups.

Fixed-effect longitudinal models [44] examined predictors of time spent in: a) walking; b) household activities; c) lower risk of knee injury activity; and d) higher risk of knee injury activity. To disentangle temporal interrelationships among perceptions of a fear of re-injury and personal importance of exercise with the total hours spent in the different activities, psychological perceptions at an earlier wave were modeled on subsequent hours spent in the different categories of activity. This enabled a better understanding of potential causal relationships. To further disentangle effects of within-person and between-person changes of a fear of re-injury and personal importance of exercise, a personal-mean centering approach was used in the longitudinal models [45–48] and included the individual respondent mean for a fear of re-injury and personal importance of exercise, as well as within-person changes in the mean across time. Analyses controlled for age, gender, BMI, pain, and sports and recreational activity limitations. Data analyses used SAS V9.2. Longitudinal modeling was estimated using the PROC MIXED procedure with an unspecified covariance structure (TYPE = UN).

## Results

At baseline, there were 121 participants, 77 men (63.6%) and 44 women ranging in age from 18–40 years (mean = 27.6 years; SD = 6.2) (Table 2). Over half of participants (53.8%) reported injuries from soccer, skiing, and basketball; 19% reported injuries as part of college/university teams; and the remainder of participants were recreational (53.7%), amateur (19.8%), and professional or minor professional (7.4%) athletes. Overall BMI remained constant over time. Perceptions of a fear of re-injury and personal importance of exercise were not significantly correlated at baseline or at any follow-up (r values ranged from −0.17 to 0.11, all p > .10). At the Year three post-surgical follow-up, 97 participants (80.1%) remained in the study. However, two participants were excluded from analyses because of missing data. Those dropping out of the study did not return for follow-up appointments after repeated attempts to contact them (Year one, n = 3; Year two, n = 4; Year three, n = 5) or had moved and were unable to travel to follow-up appointments (Year two, n = 1; Year 3, n = 11).

**Table 2:** **Means, standard deviations and 95% confidence intervals of demographic, health, fear of re-injury, personal importance of exercise and time spent in physical activities at baseline and Year 1 to 3 follow-up**

	Pre-ACL surgery baseline (n = 121)	Year 1 follow-up (n = 115)	Year 2 follow-up (n = 107)	Year 3 follow-up (n = 95)
	Mean (95% CI)	Mean (95% CI)	Mean (95% CI)	Mean (95% CI)
Age in years (SD)	27.6 (6.2)			
(range 18–40 yrs)				
Causes of initial injury (n, %)				
Soccer	33 (27.3%)			
Basketball	17 (14.1%)			
Skiing	15 (12.4%)			
Ultimate frisbee/racquet	11 (9.1%)			
Sports				
Football/Rugby	9 (7.4%)			
Baseball/Softball	8 (6.6%)			
Volleyball	6 (5.0%)			
Hockey	5 (4.1%)			
Other	17 (14.0%)			
Gender N (%)				
Male	77 (63.6)	73 (63.5)	67 (62.6)	59 (62.1)
Female	44 (36.4)	42 (36.5)	40 (37.4)	36 (37.9)
Body mass index (BMI)	24.8 (24.2, 25.4)	25.0 (24.3, 25.6)	25.1 (24.4, 25.7)	25.0 (24.2, 25.7)
Pain	66.9 (64.3, 69.6)	87.5 (85.6, 89.5)	91.8 (90.1, 93.5)	91.9^a^ (89.8, 94.1)
Sports and recreation activity limitations	10.8 (9.0, 12.5)	61.2 (56.6, 65.7)	74.3(70.0, 78.6)	78.7^a^(74.5, 82.8)
Fear of re-injury	77.2(73.0, 81.4)	49.0(43.0, 54.9)	38.6(32.4, 44.7)	32.6^a^(26.2, 39.1)
Personal importance of exercise	38.2(37.1, 39.3)	36.0(34.7, 37.4)	36.4(35.0, 37.8)	36.2(34.9, 37.6)
Physical activity				
Walking	482.6(418.8, 546.5)	436.0(358.5, 513.4)	418.1(365.8, 470.4)	452.6(378.7, 526.5)
Household activities	281.2(235.0, 327.4)	285.5(239.4, 331.6)	293.2(256.0, 330.4)	320.6(273.8, 367.5)
Lower risk for knee injury	311.5(265.0, 358.1)	201.6(165.8, 237.5)	246.4(205.6, 287.1)	214.3^b^(182.8, 245.8)
Higher risk for knee injury	192.6(156.2, 229.0)	38.5(24.4, 52.7)	110.3(69.5, 151.2)	91.8^a^(58.1, 125.4)
Physical activities				

Note: ^a^Indicates a significant difference between each wave of data (p < .05), except Year 2 and Year 3;

^b^Indicates a significant differences (p < .05) between baseline data and Year 1, Year 2 and Year 3 follow-ups.

Similar patterns of change emerged in pain, activity limitations, and fears of re-injury (Table 2). Scores were greatest at baseline (pre-surgery). The recall period for these variables largely captured post-injury experiences. Mean levels of pain, sports and recreation activity limitations and a fear of re-injury significantly decreased from baseline to Year one and from Year one to Year two. Reports of pain at Year two and Year three were low and not significantly different. Similarly, sports and recreation activity limitations and perceived fear of re-injury were lower at Years two to Year three with no further significant changes. Perceptions of the personal importance of exercise remained relatively constant over time with no significant mean changes across data collection waves.

Time spent walking and in household activities did not significantly differ from pre-surgery through Year one to three post-surgery follow-ups (Table 2). In general, respondents spent greater amounts of time in lower risk of knee injury activities than higher-risk of knee injury activities. Time spent was greatest at baseline. This is not surprising as baseline activity captured activity in the previous 12 months, which was largely pre ACL injury. The average time spent in lower risk for knee injury activity was lowest at the Year one follow-up. Surprisingly, time spent in these activities did not significantly increase at Year two and three follow-ups resulting in time spent in lower risk of knee injury physical activity being significantly lower Years one to three compared to baseline. Time spent in higher risk of knee injury physical activity was significantly lower at the Year one follow-up than all other waves of data collection. Although levels of higher risk for knee injury activity significantly increased at Years two and three, they remained significantly lower than pre-surgery (baseline) levels.

Longitudinal analyses did not reveal any factor to be significantly associated with walking (Table 3). Older participants and those whose personal importance of exercise scores increased over time spent significantly more time in household activities. Age was also associated with lower and higher risk of knee injury physical activity. Younger respondents spent significantly more time in these activities. Mean fear of re-injury scores did not significantly predict time spent in any type of activity. However, respondents who reported a greater decrease in their fear of re-injury over time spent significantly more time in higher risk of knee injury activities. Participants who reported greater importance of exercise spent significantly more time in both lower and higher risk of knee injury activities. Every 1 point increase on the scale measuring personal importance of exercise was associated with over twelve more hours of lower risk activities (12.47) and five more hours of higher risk activities (5.45).

**Table 3:** **Longitudinal mixed model analyses examining the association of demographic, pain, sports and recreation activity limitations, fear of re-injury and personal importance of exercise on subsequent time spent in walking, household activities, lower and higher risk for knee injury physical activities.**

	Walking	Household activities	Lower risk for knee injury physical activities	Higher risk for knee injury physical activities
	β (SE)	β (SE)	β (SE)	β (SE)
Age	−2.64 (3.98)	5.49 (2.55)*	−5.78 (2.08)**	−2.58 (0.96)**
Gender	1.39 (55.41)	48.86 (35.56)	−0.81 (29.84)	−21.93 (13.91)
Body mass index	−1.81 (7.13)	−3.44 (4.51)	4.83 (3.62)	0.75 (1.77)
Pain	−0.46 (1.82)	0.30 (1.11)	1.23 (0.70)	0.47 (0.36)
Sports and recreation activity limitations	0.94 (0.98)	1.10 (0.60)	−0.12 (0.37)	0.43 (0.27)
Mean fear of re-injury	−0.29 (1.15)	0.82 (0.74)	−0.76 (0.59)	−0.27 (0.29)
Change in fear of re-injury	0.66 (0.96)	1.04 (0.56)	−024 (0.35)	−0.72 (0.22)**
Mean personal importance of exercise	3.68 (4.04)	−2.37 (2.59)	10.75 (2.23)**	3.84 (1.02)**
Change in personal importance of exercise	2.46 (6.01)	11.67 (3.52)**	0.62 (2.28)	−0.28 (1.43)

Note: β = standardized regression coefficient; SE = standard errors; *p < .05; **p < .01

Coefficients were estimated using REML under PROC MIXED, SAS 9.2.

To further describe time spent in lower and higher risk of injury activities, we categorized Year three and baseline activity levels into four groups (Table 4). The groups were created using Year three data and described individuals with: 1) low fear of re-injury/low personal importance of exercise (n = 33); 2) low fear of re-injury/high personal importance of exercise (n = 25); 3) high fear of re-injury/low personal importance of exercise (n = 13); and 4) high fear of re-injury/high personal importance of exercise (n = 20). Regardless of their fear of re-injury (low or high), respondents who were low on perceived importance of exercise had reduced time spent in higher risk of knee injury activities by over 60% at Year three compared to baseline (58.8 hours (SD = 60.0) versus 165.1 hours (SD = 185.9); 64.1 hours (SD = 80.6) versus 169.4 hours (SD = 148.2)). Fear of re-injury did not appreciably change activity levels among respondents with high personal importance of exercise at Year three. These respondents generally spent more than twice as much time in lower risk of knee injury activities than higher risk of knee injury activities. Their activity did not return to baseline levels for either lower or higher risk activities.

**Table 4:** **Mean hours spent in lower and higher risk for knee injury activities for respondents with low/high fear of re-injury and low/high personal importance of exercise at the Year 3 follow-up compared to baseline**

		Low personal importance of exercise		High personal importance of exercise	
		Mean (SD)		Mean (SD)	
		Baseline	Year 3	Baseline	Year 3
**Low fear of re-injury**	Lower risk of knee injury activities	270.4(264.7)	199.8(169.6)	348.5(226.6)	234.7(137.6)
	Higher risk of knee injury activities	165.1(185.9)	58.8(60.0)	209.7(237.9)	115.9(119.0)
**High fear of re-injury**	Lower risk of knee injury activities	166.8(135.3)	129.0(112.5)	443.4(270.6)	283.8(156.1)
	Higher risk of knee injury activities	169.4(148.2)	64.1(80.6)	155.8(145.3)	128.7(319.1)

Note: SD = standard deviation.

Note: Low fear of re-injury/low personal importance of exercise n = 33.

Low fear of re-injury/high personal importance of exercise n = 25.

High fear of re-injury/low personal importance of exercise n = 13.

High fear of re-injury/high personal importance of exercise n = 20.

## Discussion

This study examined the extent to which individuals returned to physical activity over three-years following ACL reconstruction and the association of psychological perceptions like a fear of re-injury and the personal importance of exercise with the type and extent of physical activity. The research adds to existing knowledge by longitudinally examining diverse types of activity instead of focusing solely on the sport where an ACL injury occurred. Activities included walking, household activities, and sports and recreational activities that may pose lower or higher risks for knee re-injury. Findings indicated that walking and household activities were not significantly changed by an ACL injury or surgery. However, the time spent in sports and recreational activities, regardless of the potential risk for knee re-injury, was diminished and did not return to pre-injury levels three years post-surgery. Greater time spent in lower and higher risk of knee injury activities could be explained by decreases in fears of re-injury and positive perceptions of exercise as important to one’s self-identity. The findings highlight that fears of re-injury often change over time and that the perceived value of activities should be considered in research and interventions.

Similar to other studies, patients reported minimal pain and high levels of knee function post-recovery from surgery. With little variability in pain and function, it’s not surprising that these factors were not related to time spent in activities. However, other studies with a greater range of functional limitations have demonstrated the importance of pain and function to activity levels [1, 3, 7, 12].

Age was significantly associated with time spent in activities. Younger respondents reported more time in lower and higher risk of knee injury activities and older participants reported more time spent in household activities. Given age similarities in pain, function and psychological perceptions and that the participants were relatively young, age differences may reflect changes to role demands and time available for activities. Ages 18–40 years are a period of considerable transition in role responsibilities. Individuals often work full-time and take on increased responsibilities for family and household chores. Previous studies have highlighted social roles and age transitions to understand the impact of health on activity participation [49–52]. Yet, normative changes in physical activity have not been examined as a factor explaining the failure to return to baseline activity levels post ACL reconstruction. Future studies would benefit from examining demographic variables like employment, marital status, and number of children, as well as respondents’ perceptions of the time they have available for sports and exercise to further understand whether lower levels of engagement in activity is related to role demands.

The longitudinal design of this study helped better understand perceived fear of re-injury. Previous studies show that fears are associated with activity levels [1–3, 11, 14, 17, 50]. Yet, fears of re-injury were not significant in predicting activity levels longitudinally. Instead, changes in perceptions, particularly greater decreases in fears were related to greater levels of higher risk of knee injury activities. These findings provide more precision in understanding the importance of a fear of re-injury in ACL reconstruction. However, additional studies would be beneficial in gaining greater depth of understanding, especially using multi-item assessments [53] that could provide insight into why some athletes report greater reductions in fears; whether intervention efforts can reduce fears and increase activity levels; and whether increases in activities that are higher risks for knee injuries are desirable in athletes.

Also predictive of activity was the personal importance of exercise. Greater importance at any one time-point was significantly associated with more time spent in lower and higher risk of knee injury activities at later time-points. This perception was unrelated to fears of re-injury. Few studies have looked at attitudes toward exercise at an individual-difference or personality level [4], although interest is increasing with new measures examining personal importance of sport [54]. Additional descriptive information that examined activities for individuals who were low and high on personal importance of exercise further highlighted the findings. Individuals for whom exercise was not as important spent about half the hours in higher risk of injury activities than those who reported high importance of exercise. Although activity levels did not return to baseline, the findings point to differences in the value individuals place on exercise as critical in understanding returning to and sustaining sports and activity [1–4]. Of interest is what this means for future outcomes and the potential for re-injury or progression of joint damage, including the development of OA. Currently, data are lacking about the optimum level of activity, especially for higher risk of knee injury activities, following surgery. It’s not clear whether individuals with higher personal importance of exercise might be more likely to ignore clinical recommendations or engage in activities to the extent that they may do additional damage. Longitudinal research is needed to examine these issues.

Several limitations need to be acknowledged in this research. Although activities were divided into lower and higher risk for knee injury and this helped understand subsequent activity levels, categorization of activities may not always be clear and may obscure other complexities related to the activities (e.g., skill levels). Moreover, although participants were followed for three years post-surgery, additional time to monitor activity levels and assess changes to joint structures and function is needed to explain why participants did not return to lower risk for injury activities and to assess psychological perceptions in greater detail. Data assessing objective levels of activity would also be beneficial as recall or reporting biases may have influenced physical activity reports. Finally, as noted previously, it would be useful to include new psychological measures assessing fear of re-injury at a multi-level, and sport-specific perceptions of importance, as well as a greater range of demographic variables to better understand the role of activity within the broader context of an individual’s roles and responsibilities.

## Conclusions

This study highlights the importance of changes in fears of re-injury and the personal importance of exercise when predicting subsequent activity levels following ACL reconstructive surgery. It shows that, although some activities like walking and household chores are largely unaffected by ACL injuries and reconstruction, many individuals do not return to previous activity levels, even for lower risk of knee injury activities. Moreover, changes in fears of re-injury were not related to all types of activity, but only higher risk of knee injury activities. This suggests that fears may not always be a barrier to activity. Finally, age differences in activity levels may reflect changes in normative role demands (e.g., more time needed for employment) and should be examined in greater detail, as well as controlled for in longitudinal analyses. These findings may be helpful in clinical and community settings to develop interventions aimed at better enabling individuals to make decisions about participation in activities after injuries.
